# Evaluation of Permeability Recovery in Precast Concrete with Hybrid Capsules Using Constant-Head Permeability Test as Smart Construction Material

**DOI:** 10.3390/ma18020220

**Published:** 2025-01-07

**Authors:** Sung Rok Oh, Yong Jic Kim

**Affiliations:** 1 R&D Team, Newjust Co., Ltd., Gwangmyeong 14348, Republic of Korea; 2 Division of Smart Construction and Environmental Engineering, Daejin University, Pocheon 11159, Republic of Korea

**Keywords:** precast concrete, self-healing technology, hybrid capsules, crack healing efficiency, smart city infrastructure

## Abstract

This study investigates the quality characteristics and healing performance of precast concrete incorporating self-healing technology, with the aim of supporting smart city implementation. To enhance the self-healing capabilities of concrete, hybrid self-healing capsules, combining solid capsules and liquid capsules, were utilized, and their applicability and practicality were assessed. The findings revealed that incorporating hybrid self-healing capsules into precast concrete resulted in a reduction in slump by up to 14% and air content by up to 9%. Furthermore, the addition of hybrid capsules led to a maximum reduction in compressive strength of 16% and flexural strength of 18% at 28 days, while demonstrating an increase in healing efficiency as the capsule content increased. The results also indicated that the use of hybrid capsules enhanced the healing efficiency by approximately 16%, 25%, and 32% for mixing ratios of 1%, 3%, and 5%, respectively, with the overall healing efficiency ranging between 75% and 90%. Additionally, the interaction between the hybrid capsules and natural healing mechanisms promoted crystal formation around cracks, thereby significantly improving the long-term durability of the concrete.

## 1. Introduction

Recently, traditional cast-in-place reinforced concrete structures have shown limitations in quality assurance due to the influence of external factors during formwork installation and concrete pouring. Additionally, the manual nature of these construction processes leads to an increased risk of poor construction quality and safety incidents. As a result, construction costs rise, and project durations extend [1,2]. In response to these challenges, the precast concrete method has emerged as a viable alternative. It involves pre-manufacturing and storing complete or semi-finished products in a controlled plant environment [3,4]. When needed, these components are assembled on-site using lifting equipment. This approach simplifies on-site work and enhances efficiency compared to the traditional cast-in-place method, allowing for reduced on-site activities and ensuring the inherent high quality of reinforced concrete structures [5,6,7,8].

The use of precast concrete is particularly advantageous for the production of multifunctional concrete needed for smart city implementation. This is because smart concrete intended for smart city applications integrates technologies such as sensors, wireless communication, and data analytics to enable real-time monitoring of structures. Ultimately, these technologies aim to detect and predict cracks in concrete, allowing for preventive maintenance by identifying stress or vibrations in advance or for post-repair of existing cracks. While various methods are available to address cracking, recent advancements in self-healing technologies offer promising solutions from a material perspective.

Self-healing technologies focus on reducing maintenance costs and extending the service life of structures by maximizing their intrinsic ability to autonomously repair internal cracks. These technologies can prevent structural damage caused by factors such as shrinkage cracking, carbonation, and freeze–thaw cycles [9,10]. There are several types of self-healing materials, including capsules, bacteria, and organic–inorganic composites. Capsules, however, offer a unique advantage in that the healing agent is protected by a capsule membrane, which maintains its effectiveness over both short and long periods [11,12,13,14]. The mechanism involves the rupture of capsules when cracks occur, releasing the healing agent into the crack zone, where it reacts to seal the crack. Capsules are categorized based on size (nano, micro, and macro), and they can also be classified according to the phase of the healing agent. Liquid-phase and solid-phase capsules function differently, with variations in the reaction products and healing methods, leading to distinct advantages and limitations.

Most previous studies focused on using each type of capsule independently. However, recent research has explored hybrid capsules that combine different types of healing agents [15,16]. Hybrid capsules enhance crack healing by emphasizing the strengths of each material while compensating for their weaknesses.

In recent decades, research on the self-healing performance of concrete has been actively conducted. However, standardized testing methods, protocols, criteria, and indices for evaluating self-healing performance have not yet been fully established. The evaluation of self-sealing performance, which is closely related to durability, is typically conducted using permeability tests that assess self-healing performance by measuring the amount of water passing through cracks. Nonetheless, the lack of standardization in permeability testing has resulted in significant variability in test outcomes, even for specimens with similar crack widths. Crack width measurement, a critical factor in evaluating self-healing performance, is commonly performed using optical microscopy or strain gauges. However, these methods are prone to errors caused by positional variations and often fail to reflect the actual internal crack widths of specimens. To address these issues, this study adopts a constant-head permeability test to measure the water permeability of crack-induced specimens. This complements the hybrid capsule approach by providing a robust evaluation method for durability performance. The relationships between crack width and water flow rate were analyzed to evaluate the correlation between accurate initial crack width measurements and self-healing performance [17,18,19,20].

Therefore, this study aims to manufacture precast concrete using hybrid capsules as a key element for smart city implementation and evaluate its quality characteristics and durability healing performance. Specifically, the durability healing performance was assessed by evaluating permeability recovery as cracks were healed, using a constant-head permeability test.

The findings of this study emphasize the importance of sustainable building materials in constructing smart cities and propose a novel approach for establishing efficient and sustainable urban infrastructure in future cities. Additionally, this research seeks to provide foundational data for the innovative development and application of smart city technologies.

## 2. Materials and Test Methods

### 2.1. Hybrid Capsules

**Figure 1:**
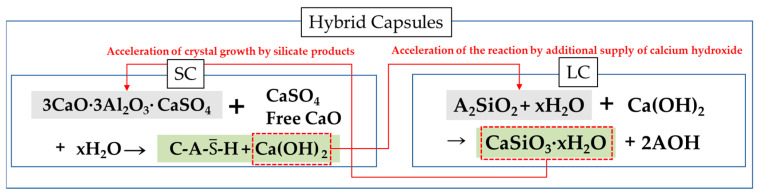
Hybrid capsule mechanism.

Hybrid capsules (HCs) are designed to compensate for the individual limitations of solid-phase capsules (SCs) and liquid-phase capsules (LCs) by combining both types. This combination allows for a particle size distribution adjustment effect due to the differences in particle sizes between SCs and LCs. In this hybrid mechanism, calcium hydroxide produced by SCs acts as a promoter for the reaction of LCs, while silicates released from microcapsules can also serve as a promoter for SC reactions. Moreover, for LCs, which are susceptible to moisture, even if the healing material dissolves in water, it can still promote the surrounding SC reaction. This complementary action emphasizes the strengths of each type while compensating for their weaknesses. The mechanism of hybrid capsules (HCs) is illustrated in Figure 1.

#### 2.1.1. Solid Capsules

The solid capsules (SCs) used in this study were composed of a mixture of inorganic materials: calcium sulfoaluminate (CSA) as an expansive agent and anhydrous gypsum (CaSO_4_). CSA, upon hydration, produces needle-like ettringite crystals, while CaSO_4_ promotes crystal growth and forms hexagonal plate-like crystals. The healing reactions of CSA and CaSO_4_ fill crack regions through the crystallization of anhydrous gypsum and achieve a wedging effect through the expansion of CSA crystals [21].

To manufacture the core material for the SCs, powdered raw materials were agglomerated using a coagulant to achieve a specific particle size. It is crucial to use a coagulant with minimal moisture content to prevent premature reactions during the agglomeration process [12]. Furthermore, the coagulant should provide adequate viscosity for particle agglomeration, and thus a urethane-based coagulant was employed in this study. The agglomerated core material, once formed, reacts with moisture, and it is essential to prevent premature reactions with the mixing water when incorporated into concrete. To enhance the surface strength of the core material, it was coated with a membrane material.

**Figure 2:**
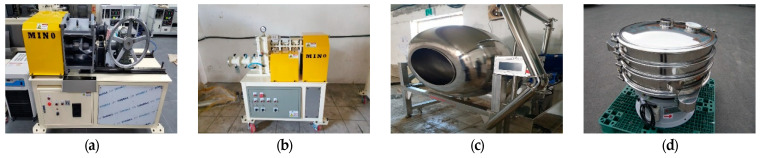
Equipment of solid capsules: (**a**) Mixer, (**b**) Core manufacturing, (**c**) Film coating, (**d**) Sorting.

Table 1 presents the chemical composition of the expansive materials. The membrane material used in this study was polyurethane, to which toluene was added to control its viscosity. There are several steps in the process of producing the base material for SCs: the material is mixed (Figure 2a), granulated (Figure 2b), coated with a protective membrane (Figure 2c), and finally graded by size (Figure 2d).

The SCs were manufactured to sizes ranging from 600 to 2360 μm. Based on previous studies [13,15], an optimal size was selected, and only the SCs with a size of 850 μm were used in this study.

#### 2.1.2. Liquid Capsules

The liquid capsules (LCs) used in this study were composed of inorganic silicate-based materials. These silicate-based materials included a mixture of potassium silicate (PS), sodium silicate (SS), and lithium silicate (LS). Table 2 presents the chemical composition of the silicate-based inorganic materials used in the cores of LCs. The reaction mechanism of these silicate-based materials involves their interaction with calcium hydroxide (Ca(OH)_2_), a cement hydration product. This reaction produces calcium silicate hydrate (CaSiO_3_·xH_2_O) and releases strongly alkaline metal ions such as K^+^, Na^+^, and Li^+^. The healing process occurs as the calcium silicate hydrate gels and fills the crack region, while the strongly alkaline metal ions promote the additional hydration of unreacted cement around the cracks. These reaction products not only heal cracks but also densify the internal structure of concrete, helping to maintain or restore the high pH of the alkaline environment within the concrete [22].

**Figure 3:**
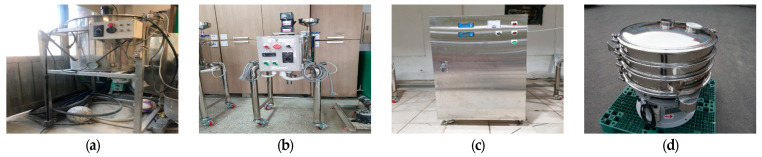
Equipment of liquid capsules: (**a**) Synthetic liquid manufacturing, (**b**) Film forming, (**c**) pH automatic control, (**d**) Sorting.

The membrane material for the LCs consisted of conventional polyurethane (PU), urea, and formaldehyde. To control the viscosity of PU, toluene was added at approximately 60% dissolution [22]. The synthesis of the LCs involved multiple steps using the equipment shown in Figure 2. First, the core material was synthesized using the apparatus in Figure 3a, followed by membrane formation using the equipment in Figure 3b. During this process, pH adjustment was conducted using the device in Figure 3c. Finally, size classification was performed with the apparatus in Figure 3d to complete the manufacturing process. The LCs were manufactured to sizes ranging from 30 to 350 μm. Based on previous research [22], the optimal particle size was selected within the range of 160 to 350 μm for this study.

Table 3 shows the physical properties of the silicate-based materials used in the core of the LCs.

### 2.2. Materials

The cement used for the precast concrete was Type I ordinary Portland cement (OPC). The aggregates used in the experiment consisted of river sand (S) as the fine aggregate and crushed gravel (G) as the coarse aggregate, with the maximum size of coarse aggregate being 25 mm. The chemical admixture (ad) used in the experiment was a polycarboxylate-based high-range water reducer produced by a domestic company, H Corporation. This admixture was selected to ensure the dispersion of the capsules and to maintain workability.

#### Mix Proportions

Recently, the Korean Design Standard (KDS) and the Korean Construction Specification (KCS) were integrated into a unified system known as the 2021 Construction Standards Code. Therefore, the mix design for the precast concrete in this study followed the KCS 14 20 52 Precast Concrete (2021) [23] specifications. For other aspects not covered under these specifications, the design was based on KCS 14 20 10 General Concrete (2021) [24]. The mix design criteria for the precast concrete used in this study were based on those applied to water treatment structures such as concrete water tanks.

Table 4 provides the specifications for concrete production, and Table 5 shows the detailed mix proportions. For the HCs, four different mixing levels were applied (0%, 1%, 3%, and 5%) based on the mass of the fine aggregate. The healing performance of the concrete was evaluated by isolating the coarse aggregates to focus on its healing capabilities.

### 2.3. Evaluation Methods

#### 2.3.1. Basic Quality Performance

The basic quality performance of the precast concrete was evaluated in terms of flow and mechanical performance. Flow performance was assessed by measuring the slump and air content, following the KS F 2402 standard, “Test Method for Slump of Portland Cement Concrete” [25] and the KS F 2421 standard, “Test Method for Air Content of Fresh Concrete by Pressure Method” [26].

For mechanical performance, compressive strength was evaluated in accordance with KS F 2405, “Test Method for Compressive Strength of Concrete” [27].

#### 2.3.2. Constant-Head Permeability Test

**Figure 4:**
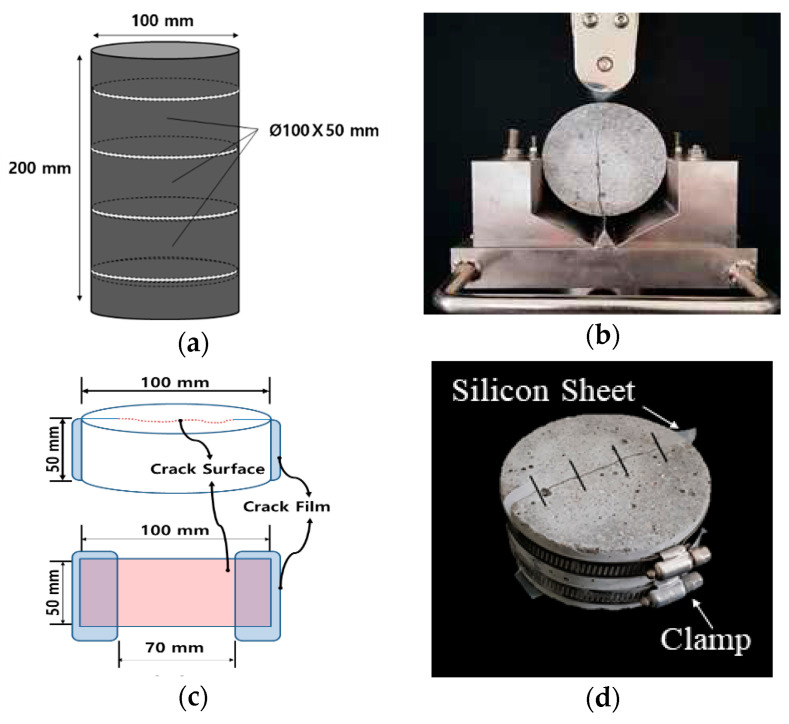
Preparation of cracked specimens: (**a**) slicing into three-disc specimens, (**b**) specimen splitting, (**c**) crack induction, and (**d**) binding together of specimens with two steel bands.

A constant-head permeability test was adopted to measure the water flow rate of the crack-induced specimen and to evaluate its self-healing performance [17,28]. For this test, cracked specimens were prepared following several steps. First, mortar cylinders (Ø100 × 200 mm) were cast, as shown in Figure 4a. After 24 h, the cylinders were demolded and cured in a water bath at 20 °C until the desired age for crack induction was reached.

Once the cracking age was achieved, each cylinder was sliced into three-disc specimens (Ø100 × 50 mm) and then split into two semi-circular sections, as shown in Figure 4b. A flexible silicone rubber sheet of varying thickness was attached to both ends of the cracked sections to induce a crack with a specified width, as shown in Figure 4c. The actual lengths of the cracks were approximately 70 mm. Finally, the split specimens were bound together using stainless steel bands to maintain the desired crack width, as shown in Figure 4d. For each specimen, cracks were induced at 28 and 91 days, with crack widths ranging from 0.2 to 0.25 mm. After the specimens were prepared, the widths and lengths of the cracks were measured using a microscope (EGVM-35M, EG Tech, Anyang, Republic of Korea).

**Figure 5:**
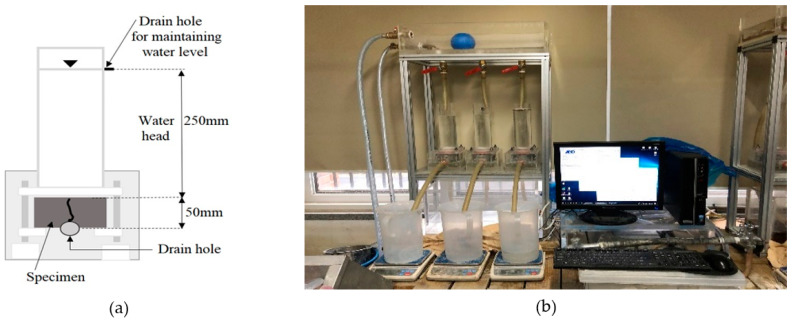
Water permeability test apparatus: (**a**) schematic diagram and (**b**) test setup.

During the healing period, the cracked specimens were cured in a water bath maintained at 20 °C, as there is no specific standard for water temperature during healing [28,29]. A water permeability test was conducted after healing periods of 0, 7, 14, 21, and 28 days. Figure 5a shows the schematic diagram of the water permeability test setup, and Figure 5b shows the test apparatus used for the cracked disc specimens. The amount of water discharged from the equipment was measured for 7 min after the water head and flow stabilized. The water flow rate, expressed in units of mL/(mm·min), was calculated by dividing the total discharged water by the test duration (minutes) and crack length (mm).

The healing index, *SH_q_*, was calculated based on the reduction in water flow rate using the following equation [28,29,30,31]:

(1) $${SH}_{q}=\left[1-\frac{q(t)}{q0}\right]\times 100\left(\%\right)$$

where *q*0 is the initial water flow rate measured immediately after cracking the specimen, without any healing effect, and *q*(*t*) is the water flow rate after a healing period, *t*.

## 3. Results and Discussion

### 3.1. Fundamental Properties

**Figure 6:**
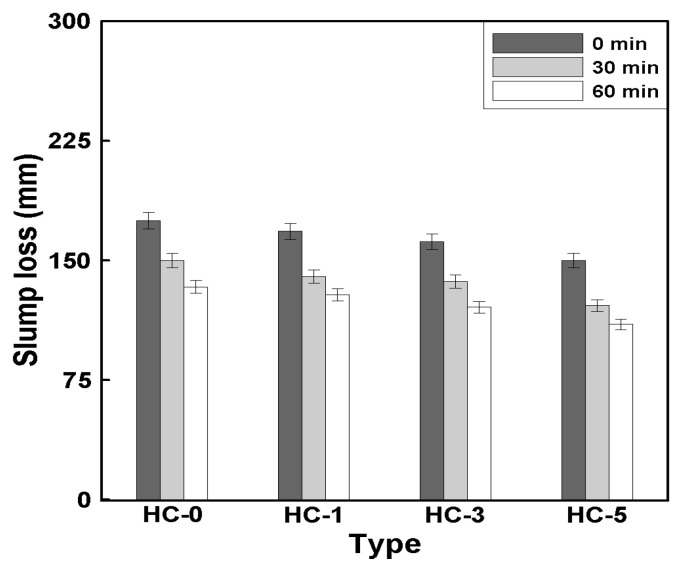
Relationship between HC mixing rate and slump loss.

Figure 6 shows the relationship between the HC mixing ratio and slump loss, where a decreasing trend in slump was observed with the addition of HCs. This indicates that as the mixing ratio increased, the slump value decreased. The slump of HC-0 was approximately 175 mm, while the slumps of HC-1, HC-3, and HC-5 decreased by about 4%, 8%, and 14%, respectively, compared to HC-0, as the HC mixing ratio increased by 2%.

These findings align with previous studies [12,15,22], which reported a slump loss of approximately 10% during the mixing process involving HCs. The primary reason for this loss has been attributed to friction between the concrete’s constituent aggregates and the mixer blades. Therefore, it can be concluded that the observed slump reduction was likely due to these frictional effects during the mixing process.

**Figure 7:**
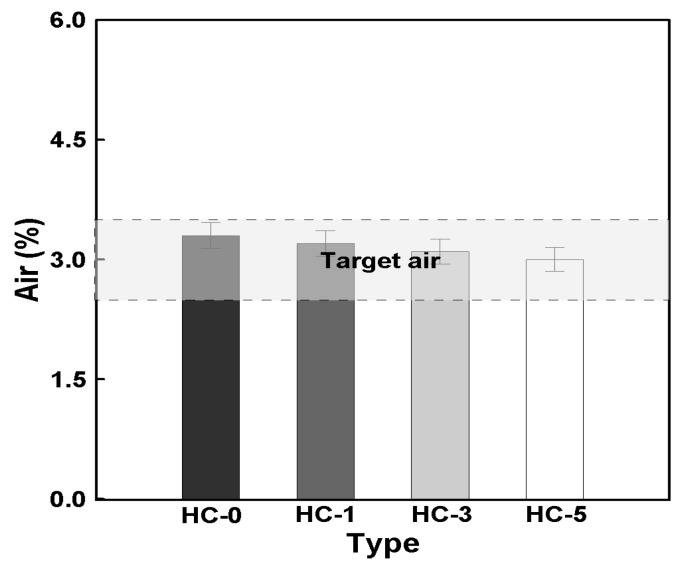
Relationship between HC mixing rate and air.

Figure 7 shows the relationship between the HC mixing ratio and air content. The air content of HC-0 was approximately 3.3%, while those of HC-1, HC-3, and HC-5 decreased by around 3%, 6%, and 9%, respectively. Additionally, with a 2% increase in the HC mixing ratio, a proportional decrease of approximately 3% was observed.

Although there was a slight reduction in air content with increasing HC ratios, the decrease remained within the margin of error and was not significant. Therefore, it can be concluded that regardless of the HC mixing ratio, the air content remained at an equivalent level. These results suggest that the addition of HCs did not significantly affect the air content of the concrete.

**Figure 8:**
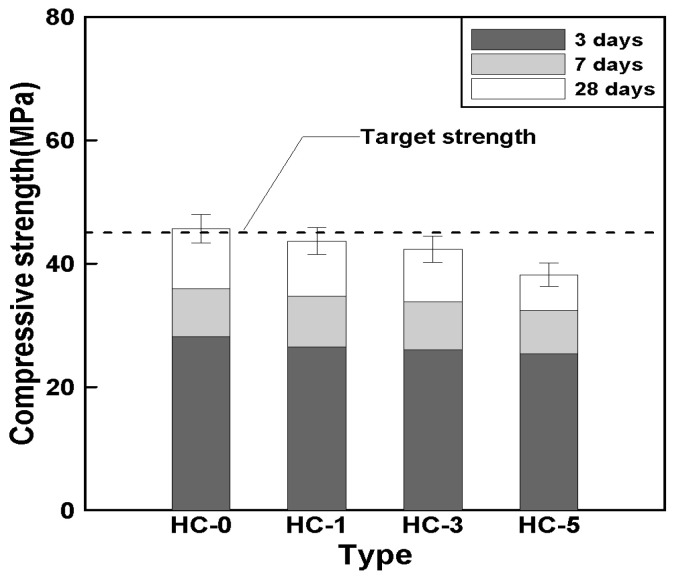
Relationship between HC mixing ratio and compressive strength.

Figure 8 illustrates the relationship between the HC mixing ratio and compressive strength over time. The 28-day compressive strength of HC-0 was approximately 45.6 MPa. In comparison, the 28-day compressive strengths of HC-1, HC-3, and HC-5 showed reductions of around 4%, 7%, and 16%, respectively, compared to HC-0.

These findings align with previous studies [12,15,22], which reported that the compressive strength decreases as the HC mixing ratio increases. This reduction is attributed to the relatively lower particle strength of HCs compared to the concrete’s binder and aggregate. As a result, the regions containing HCs could not sufficiently bear the applied load, leading to a reduction in overall compressive strength.

It can be concluded that the addition of HCs had a negative effect on the compressive strength of concrete, with strength reductions reaching up to 16% as the HC mixing ratio increased. However, the strength development rate over time showed similar levels regardless of the mix type, indicating that the addition of HCs did not affect the rate of strength development.

**Figure 9:**
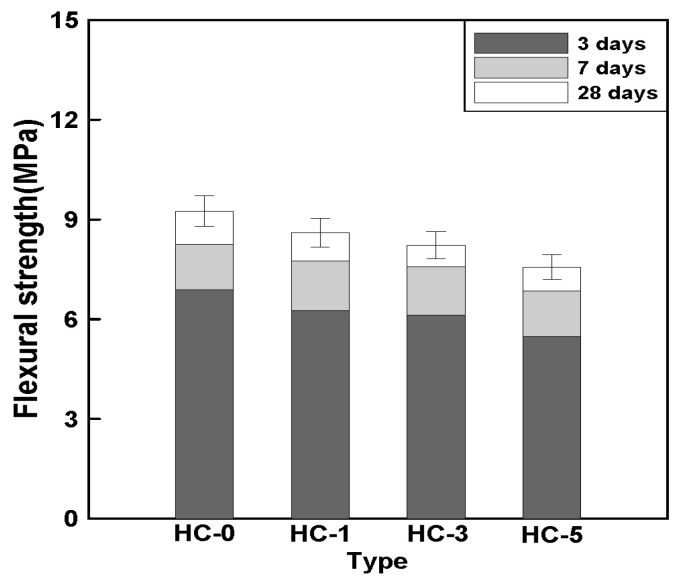
Relationship between HC mixing ratio and flexural strength.

Figure 9 illustrates the relationship between the HC mixing ratio and flexural strength over different curing periods. The 28-day flexural strength of HC-0 was approximately 9.3 MPa. In comparison, the 28-day flexural strengths of HC-1, HC-3, and HC-5 showed reductions of around 7%, 11%, and 18%, respectively, as the HC mixing ratio increased by 2%.

These results are attributed to the same factors that influenced the reduction in compressive strength. However, the more significant decrease in flexural strength can be explained by the increase in the interfacial transition zones (ITZs) associated with higher HC mixing ratios. As the proportion of HCs increased, the ITZ became more pronounced, leading to a greater reduction in flexural strength.

It can be concluded that the addition of HCs negatively impacted the flexural strength of concrete, with reductions reaching up to 18% as the HC mixing ratio increased. However, similar to the results for compressive strength, the rate of strength development over time remained consistent across all mix types, indicating that the addition of HC did not significantly influence the rate of strength gain with age.

### 3.2. Healing Performance

**Figure 10:**
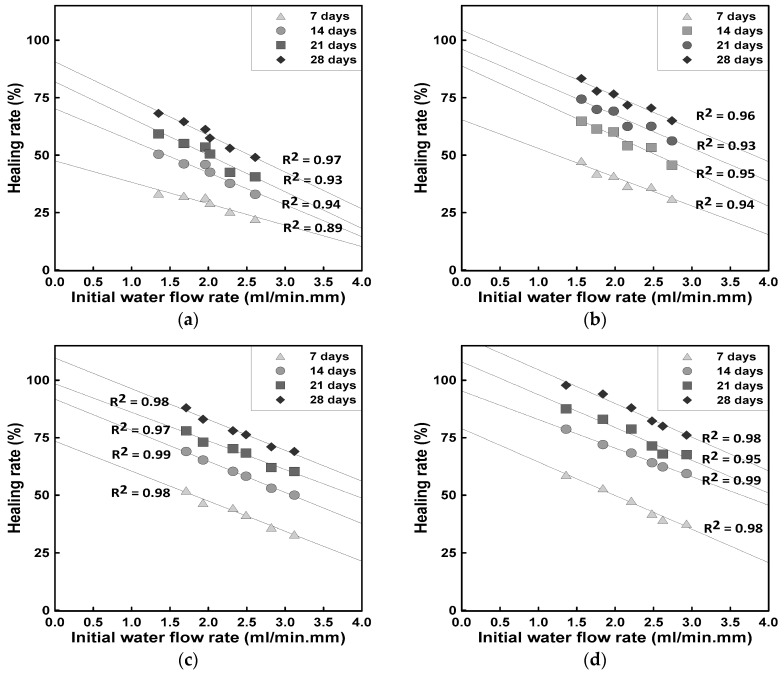
Correlation between initial permeability and healing rate according to healing age: (**a**) HC-0, (**b**) HC-1, (**c**) HC-3, (**d**) HC-5.

Figure 10 shows the relationship between the initial water permeability and healing efficiency over the healing period for HC-0, HC-1, HC-3, and HC-5. All mixes exhibited an increasing trend in healing efficiency as the healing period progressed, with the level of healing improving as the HC mixing ratio increased. This indicates that even HC-0, which did not contain hybrid capsules, possesses some degree of natural healing ability. This natural healing can be attributed to unhydrated cement particles near the cracks that formed crystals, reducing the crack width and thereby decreasing water flow.

The effectiveness of HCs lies in enhancing and promoting their natural healing capability. SCs contributed to the formation of needle-like ettringite and plate-like calcium hydroxide crystals, while LCs released silicate-based products and strongly alkaline ions. The combined effect of SCs and LCs not only complemented each other but also enhanced the existing natural healing process.

According to previous studies, an initial water flow rate of 1.0 to 1.8 mL/min·mm corresponds to a crack width of 0.3 mm. Through regression analysis of the initial water flow rate in the range of 1.0 to 1.8 mL/min·mm, the healing efficiency after 28 days of healing showed that HC-0 achieved approximately 62%, HC-1 about 78%, HC-3 about 87%, and HC-5 about 94%. The effect of HC addition resulted in an improvement of approximately 16% for HC-1, 25% for HC-3, and 32% for HC-5 compared to the natural healing of HC-0.

The healing efficiency with increasing HC mixing ratios showed a relatively higher rate for HC-5. This can be attributed to the larger quantity of healing products and an increased rate of healing due to the higher HC content. However, it should be noted that excessive HC mixing to increase healing efficiency may lead to a decline in basic quality performance. Therefore, it is essential to determine an optimal HC mixing ratio that meets the target performance requirements.

In this study, the healing efficiency was evaluated based on severe conditions involving full-depth cracks. In actual cases, where cracks typically begin as microcracks and gradually expand, sufficient healing effects can be achieved with lower HC mixing ratios. This is because cracks in real environments generally start as fine microcracks, and since HC reacts immediately upon crack formation, it not only heals the initial microcracks but also mitigates crack propagation. Thus, effective healing can be achieved with lower amounts of HC.

**Figure 11:**
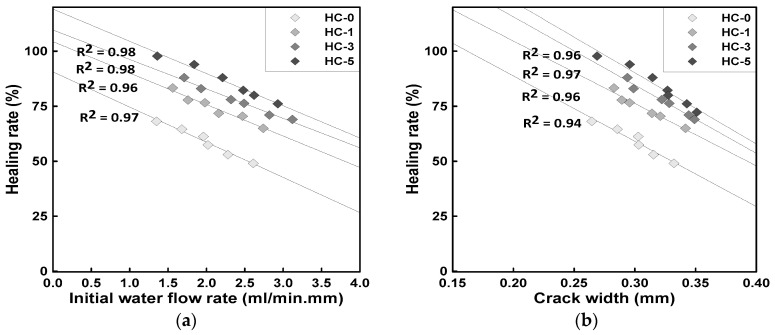
Healing rate based on a period of 28 days of healing: (**a**) Initial water flow rate by experiment, (**b**) Initial water flow rate converted to crack width.

Figure 11a illustrates the relationship between the initial water flow rate and healing efficiency after 28 days of healing, based on the experimental results presented in Figure 10. Figure 11b shows the conversion of the initial water flow rates from Figure 10a into equivalent crack widths. According to previous studies [17,31,32,33,34], the concept of equivalent crack width allows the estimation of crack widths based on the initial water flow rate.

Since only the surface crack width could be observed in the crack-induced specimens, it was challenging to estimate the full depth of the cracks within the specimens. Therefore, an analysis was conducted to predict crack widths solely based on the relationship between the initial water flow rate and the crack width.

The results in Figure 11b indicate that when the initial water flow rate corresponded to a crack width of 0.2 mm, it was approximately 0.6 mL/min·mm or lower. For a crack width of 0.25 mm, the initial water flow rate ranged from approximately 0.7 to 1.0 mL/min·mm, and for a crack width of 0.3 mm, it ranged from about 1.0 to 1.8 mL/min·mm. These findings suggest that the initial water flow rate obtained from the constant-head permeability test can be used as an indicator to estimate crack widths and evaluate the self-healing performance of the cracks.

This approach allows for a relatively intuitive evaluation by converting the experimental results from the constant-head permeability test into crack widths. It also provides a useful method for analyzing experimental results in terms of crack width, facilitating a clearer understanding of self-healing performance based on crack size.

**Figure 12:**
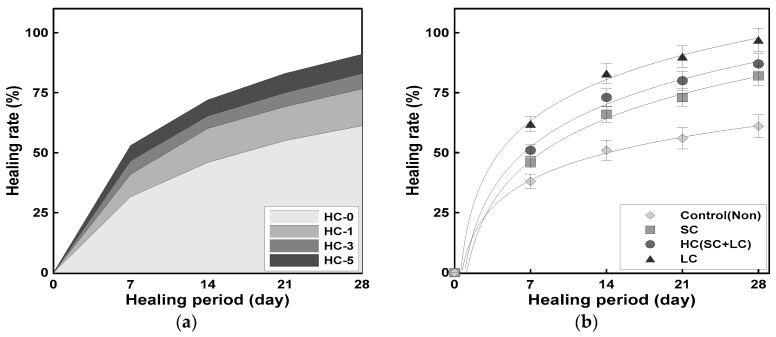
Correlation between healing time and healing rate: (**a**) healing energy (effective area) based on crack width of 0.3 mm; (**b**) comparison of healing rates of HCs, SCs, and LCs.

Figure 12a illustrates the allowable healing range for HC, specifically for a crack width of 0.3 mm. After 28 days, the healing efficiency was approximately 61% for HC-0, around 77% for HC-1, about 83% for HC-3, and nearly 91% for HC-5.

These results indicate that the healing efficiency increased as the HC mixing ratio increased. However, since this range quantitatively defines the allowable healing, it can serve as a useful reference indicator when designing self-healing systems for actual conditions.

Figure 12b presents an analysis from previous studies [13,15] that shows the relationship between the healing period and healing efficiency according to the type of capsule. This analysis was conducted to compare the effects of using LCs and SCs individually with HCs. The comparison was based on a lower healing efficiency threshold of 3%. The results in Figure 12b indicate that, over time, the healing efficiency increased for all three cases (LC, SC, and HC). After 28 days, the healing efficiency was approximately 97% for LCs, about 82% for SCs, and around 87% for HCs.

Although the healing efficiency of HCs was approximately 10% lower than that of LCs, it was about 5% higher compared to SCs. This suggests that while LCs alone demonstrated the most effective self-healing performance, they present cost challenges when used independently. Therefore, using HCs, which combine LCs and SCs, offers a balanced approach by enhancing self-healing performance while potentially reducing costs.

## 4. Conclusions

In this study, the quality characteristics and healing performance of precast concrete incorporating self-healing technology were evaluated as a key element for implementing smart cities. By manufacturing and analyzing precast concrete utilizing crack self-healing capsules, the applicability and practicality of this technology were assessed, leading to the following conclusions:

(1) The incorporation of hybrid self-healing capsules into precast concrete resulted in a reduction in slump by up to approximately 14% and a reduction in air content by up to approximately 9%.
(2) Hybrid self-healing capsules had a noticeable impact on the strength of the concrete, causing compressive strength to decrease by up to approximately 16% and flexural strength by up to approximately 18% at 28 days. Despite these reductions, healing performance improved with higher mixing ratios, with notable improvements in healing efficiency for 0.3 mm wide cracks.
(3) The addition of hybrid self-healing capsules significantly enhanced healing performance, increasing efficiency by approximately 16%, 25%, and 32% at mixing ratios of 1%, 3%, and 5%, respectively. The overall healing efficiency ranged from a minimum of 75% to a maximum of 90%.
(4) The hybrid capsules not only interact with existing natural healing mechanisms but also enhance crystal formation around cracks, thus improving the overall healing performance of the concrete. This interaction contributes to the long-term durability of concrete structures.
(5) The combination of LCs and SCs in hybrid capsules offers improved cost efficiency compared to using LCs alone, making it a more economically viable solution for implementing self-healing systems in smart cities.
(6) The initial water flow rate measured in constant-head permeability tests serves as a practical indicator for predicting and managing crack widths in field applications, providing an intuitive approach to assessing crack progression and self-healing capabilities.
(7) The precast concrete developed in this study demonstrated effective healing performance under severe conditions with full-depth cracks. In actual environments, cracks typically begin as microcracks and gradually expand, suggesting that sufficient healing effects can be achieved with lower mixing ratios.

These findings demonstrate the potential of precast concrete incorporating crack self-healing capsules as a key element in smart city implementation, and this technology is expected to contribute to the development of sustainable and safe urban infrastructure.

## Figures and Tables

**Table 1:** Chemical composition of expansion materials.

Type	%
Calcium Oxide	45–55
Calcium Sulfate, Gypsum	25–35
Calcium Sulfoaluminate	5–10
Dicalcium Aluminate, Iron Oxide	1–5

**Table 2:** Chemical compositions of silicate-based materials.

Type	Potassium Silicates	Sodium Silicates	Lithium Silicates
Specific gravity (20 °C)	1.27~1.29	≥1.38	1.15~1.20
K_2_O (%)	10.0~11.0	–	–
Na_2_O (%)	–	9.0~10.0	–
Li_2_O (%)	–	–	1.0~1.5
SiO_2_ (%)	21.5~22.5	28.0~30.0	18.0~22.0
Fe_2_O_3_ (%)	0.05	0.03	–

**Table 3:** Physical properties of silicate-based materials.

Type	Mole Fraction	Viscosity (cps, 20 °C)	Solid Content (%)
Potassium silicates	3.2~3.5	≤20	20~52
Sodium silicates	3.10~3.30	30–50	30~56
Lithium silicates	7.5~8.5	10–20	20~25

**Table 4:** Specifications of precast concrete.

Item	Required Performance
Compressive strength	45 MPa
Air	3%
Slump	170 mm
Gmax *	25 mm

Gmax *: Maximum size of coarse aggregates.

**Table 5:** Precast concrete mixing table.

Type	W/C	S/a	Unit Weight of Material (kg/m^3^)				ad (C mass×%)	HC (S vol.×%)
			W	OPC	S	G		
HC-0	39.3	49.0	165	420	862	908	0.8	0
HC-1	39.3	49.0	165	420	862	908	0.8	1
HC-3	39.3	49.0	165	420	862	908	0.8	3
HC-5	39.3	49.0	165	420	862	908	0.8	5

## Data Availability

Data are available on request due to restrictions, e.g., privacy or ethical.
